# Production and characterization of metal nanoparticles for chemical reduction in order to application in biological systems

**DOI:** 10.1186/1753-6561-8-S4-P263

**Published:** 2014-10-01

**Authors:** Wanderson Keijok, Bárbara Milaneze, Jairo Oliveira, Brunelli Peruch, Adilson Prado, Janine Bartochevis, Maria Pontes, Moises Ribeiro, Breno Nogueira, Marco Guimarães

**Affiliations:** 1 Universidade Federal do Espírito Santo (UFES), Vitória, ES, Brazil

## Background

Several studies have demonstrated the use of metallic nanomaterials with applications in the biological area with very promising results. Gold nanoparticles (AuNP's) have attracted great interest from researchers due to its electronic properties, optical, thermal, and its great potential for application in physical, chemical and biological [1].

In general, the AuNP's are prepared by chemical reduction starting from a precursor (usually HAuCl_4_) is a strong reducing agent. The shape and size of the nanoparticles depend on the synthesis process variables such as concentration of the reactants, temperature, pH, reaction time, and others. The same material composition can be determined with different physical and chemical characteristics just modifying characteristics such as size, self-organization, crystalline structure and shape, the point of the material in the nanometer range show distinct physical and chemical properties of materials on the macroscopic scale [2]. The aim of this work was to prepare and characterize gold nanoparticles using the method of chemical reduction with sodium borohydride (NaBH_4_) in order to evaluate the influence of the concentration of the reducing agent and the time of synthesis on the properties of NP's. The proposal is to use the material generated to evaluate its bactericidal effect.

## Methods

In order to evaluate the effect of variables on conversion of the reaction, as well as finding the conditions that maximized the synthesis of nanoparticles, one factorial design (3^2^) with 3 levels and 2 variables was performed. The variables evaluated were: Concentration of Reducing Agent (1.0, 2.0 and 3.0 %) and the time synthesis (5, 10, 15 min). These intervals were defined to cover most of the studies described in the literature [3,4]. To prepare the AuNP's, the reducing agent (sodium borohydride - NaBH4) was added according to experimental design gold precursor solution (HAuCl_4_ 2.5 × 10^-4^ M) at room temperature and was kept under stirring the pre defined.

AuNP's samples were collected after the synthesis step and their optical properties were evaluated by UV-vis spectrophotometer (Shimadzu). The size and morphology of Au nanoparticles were examined by transmission electron microscopy (JEM-1400, JEOL Inc., USA).

## Results and conclusions

The preparation of the material was evaluated on the basis of electronic spectra obtained and characterized by transmission electron microscopy was performed to determine the diameter and distribution. The results were analyzed by STATISTICA software 12 and showed that the concentration measured was only significant in study levels. The electron microscopy images showed a non-uniformity of the size of the generated material, probably because the character does not have borohydride stabilizer. The synthesized material will be evaluated for its bactericidal effect.
